# Sex Differences in Heat Shock Protein 72 Expression in Peripheral Blood Mononuclear Cells to Acute Exercise in the Heat

**DOI:** 10.5812/ijem.8739

**Published:** 2013-10-11

**Authors:** Trevor Gillum, Matthew Kuennen, Cheryl Gourley, Karol Dokladny, Suzanne Schneider, Pope Moseley

**Affiliations:** 1 Department of Kinesiology, California Baptist University, Riverside, USA; 2 Department of Sports and Exercise Science, West Texas A&M University, Canyon, USA; 3 Department of Health, Exercise and Sport Sciences, The University of New Mexico, Albuquerque, USA; 4 Department of Internal Medicine, The University of New Mexico, Albuquerque, USA

**Keywords:** Menstrual Phase, Thermoregulation, Endogenous antioxidants, Thermotolerance

## Abstract

**Background:**

Heat shock protein 72 (Hsp72) is responsible for maintaining critical cellular function during heat stress. Hsp72 confers thermotolerance and may play a role in heat acclimation. Animal research suggests a difference between sexes in Hsp72 expression in response to exercise, however, human data is lacking.

**Objectives:**

To determine sex differences in intracellular heat shock protein 72 (Hsp72) following exercise in the heat.

**Patients and Methods:**

Nine non-heat acclimated women with normal menstrual cycles (VO_2pk _58 ± 5 mL.kgFFM^-1^.min^-1^) and nine non-heat acclimated men (VO_2pk_ 60 ± 7 ml.kgFFM^-1^.min^-1^) completed 2 treadmill bouts at 60% VO_2pk_ for 60 min in a 42°C, 20% RH environment. Women were tested in follicular (fol) and luteal (lut) phases. The duplicate trials were separated by 12 days for men and women. Blood samples were drawn pre, immediately post, 1, and 4 hrs post-exercise.

**Results:**

Men and women differed in their Hsp72 response after exercise (time X sex X trial interaction; P < 0.05). Men increased Hsp72 after exercise more than women. Both men and women produced less Hsp72 during trial 2 compared to trial 1. Estrogen (r = 0.24; P > 0.05) and progesterone (r = 0.27, P > 0.05) concentrations were not correlated with Hsp72.

**Conclusion:**

Our findings suggest that men and women differ in their cellular stress response. Men up-regulated Hsp72 after a single bout of exercise in the heat, which persists for 12 days, suggesting an accumulation of Hsp72 which may lead to acquired cellular thermotolerance.

## 1. Background

The cellular stress response is characterized by the accumulation of intracellular heat shock proteins, including the highly heat inducible 72-kDa heat shock protein 72 (Hsp72). Hsp72 expression is linked to thermotolerance (1) in that increased Hsp72 protects cells against normally lethal increases in core temperature (2, 3). Hsp72 may protect cells by acting as an intracellular chaperone enhancing protein folding (4), inhibiting intracellular pro-inflammatory cytokine synthesis (5-8), and/or limiting cellular apoptosis (7). Males have shown an increased expression of Hsp72 in peripheral blood mononuclear cells (PBMC) (6, 7, 9, 10), skeletal muscle (11), brain (12) and liver (13) within 4 hrs after exercise or heat stress. 

Given that Hsp72 is accumulated in response to stress, it is interesting that animal studies have shown a difference in the expression of Hsp72 between sexes. Under resting conditions, basal levels of Hsp72 in cardiac and renal tissue are higher in female compared to male rats (14, 15). However, in response to running (16, 17) and hyperthermia (18), male animals are able to express higher Hsp72 than females in the gastrocnemius and cardiac tissue. In the above studies, sham treated ovariectomized females had similar post stress Hsp72 levels as males, while estrogen treated ovariectomized females showed unaltered Hsp72 levels after stress, similar to intact females. Animal studies suggest an increased basal expression of Hsp72 may reduce the cellular response to an exercise stress and thus limit the need for additional Hsp72 production after the stress (19). The mechanism behind the relationship between estrogen and the blunted intracellular Hsp72 response to stress currently is not known. It was suggested that estrogen mediates this effect through a nongenomic hormonal pathway (17). Currently, there are no human studies that have tested for possible sex differences in the cellular stress response to exercise in the heat.

## 2. Objectives

Therefore, the objective of this study was to compare the expression of Hsp72 in PBMC at rest and after exercise in the heat in men and women. We hypothesized that women would have an increased baseline expression of Hsp72, but an attenuated post exercise response compared to men.

Furthermore, we examined if women in follicular (fol) and luteal (lut) phases of the menstrual cycle differed in their amount of Hsp72 expressed in response to exercise in the heat. We hypothesized that during lut, when estrogen levels are elevated, there would be a decrease in Hsp72 at rest and after exercise compared to fol.

## 3. Materials and Methods

### 3.1. Subjects

Eighteen (9 men and 9 women) subjects completed two treadmill sessions in a hot, dry environment (42.3 ± 1°C, 22.5 ± 12% relative humidity). Men were matched with women for aerobic fitness (ml ∙ kg lean body mass ^-1 ^min ^-1 ^) and age (Table 1). None of the women were taking hormonal contraceptives and all had menstrual cycles of 28-32 days for three months prior to testing. Women were tested during the fol (day 7 ± 2) and lut (days 20 ± 1) phases of their menstrual cycle. Progesterone concentration was used to validate the presence of the luteal phase, as previously done ( 20 , 21 ). The same length of time elapsed between the two exercise trials for men and women (M: 12 ± 4 days, W: 12 ± 2 days). Each exercise trial for a given subject was conducted at the same time of day. Between exercise trials, subjects were asked to continue their normal physical activity patterns. The University of New Mexico’s Institutional Review Board approved this protocol and the subjects provided written, informed consent prior to participation. 

### 3.2. Preliminary Testing

Body composition and cardiorespiratory fitness were assessed for all subjects. Three site skinfold (Lange, Beta Technology, Santa Cruz, CA) measurements (M: chest, abdomen, thigh; W: triceps, suprailiac, thigh) were used to determine percent body fat. Each site was measured in triplicate and the mean value was used to calculate percent body fat (22). A continuous graded treadmill test in a temperate room (22-24°C, 30% RH) was used to determine VO_2peak_. VO_2peak _was assessed through open circuit spirometry (ParvoMedics, Sandy, UT) and defined as the highest 30 second value when 2 of the following criteria were met: 1) a plateau in VO_2_ (change in VO_2_ < 150 mL min^-1^) with increased workload, 2) a maximal respiratory exchange ratio greater than 1.1 and 3) heart rate greater than 95% of the age predicted maximum (220-age). VO_2 peak _was expressed per mL of fat free mass (FFM) so that the aerobic fitness of sexes could be compared.

### 3.3. Experimental Design

Each subject performed two exercise trials (treadmill running at 60% VO_2peak _for 60 min) in an environmental chamber maintained at 42°C, 20% RH. The trials for women were counterbalanced so that 4 subjects performed the first exercise bout in the lut phase while 5 subjects performed the first exercise bout in the fol phase. Plasma estrogen and progesterone values were obtained to corroborate the appropriate menstrual cycle phase.

### 3.4. Exercise Trial

Data collection took place during the Fall and Winter months (October – February) to limit the effects of heat acclimation. Subjects were instructed to avoid exercise and alcohol for 24 hrs and to avoid caffeine for 12 hrs prior to each exercise trial. Subjects were given a list of high carbohydrate foods to consume for dinner on the night before and for breakfast on the morning before each trial. Subjects were asked to consume the same foods before the two trials.

On the day of the trial, nude body weight was recorded to the nearest 0.1 kg (Seca Scale, Birmingham, UK) and urine osmolality was used to assess the subjects’ hydration status (Advanced Osmometer, Model 303, Advanced Instruments Inc, Norwood, MA). 

Core temperature (T_r_) was measured by inserting a thermistor (YSI precision 4400 Series, Yellow Springs Inc, Yellow Springs, OH) 10 cm past the anal sphincter. An intravenous catheter was inserted in an antecubital vein and kept patent by infusing 3 ml of isotonic saline every 15 min during and after the exercise trial. Samples were drawn pre, immediately post, 1 hr post and 4 hrs post exercise. 

The exercise intensity was set to elicit 60% of VO2peak. The intensity of exercise was selected such that active individuals who were not endurance trained would be able to complete the protocol. VO_2 _was measured every 15 min during exercise. The speed and grade were adjusted during the first 15 min of trial 1 to obtain a VO_2_ of 60% of VO_2peak_. This exercise level was maintained for the remainder of the exercise trial. When subjects repeated the exercise trial, the treadmill speed and grade were identical to the first exercise trial. During the first exercise trial, the subjects were allowed to drink water ad libitum. Each subject then ingested the same volume of water during their second trial. After exercise, each subject dried themselves with a towel and obtained their nude body weight. Sweat loss was calculated as the difference in pre to post weight, corrected for fluid intake. One male and one female subject were unable to complete their first 60 min exercise trial due to nausea and light headedness. When the trial was repeated, these subjects then exercised for this same duration. Hsp72 for these subjects was not anomalously higher after the first trial compared with the second. Therefore, data from these subjects are included in the analysis.

### 3.5. Blood Collection and Preparation

Three ml of blood were drawn at each time point and transferred to EDTA treated tubes. Half of the blood was aliquotted to measure hematocrit, plasma estrogen, and progesterone. The other half was used to measure Hsp72. Hematocrit was analyzed within 15 min of the blood draw and used to correct plasma protein concentrations for plasma volume changes during exercise as described elsewhere (23). After measuring hematocrit (microcentrifuge technique), the blood was centrifuged and the plasma separated and stored at -80ºC until later analysis. 

**Figure 1.**
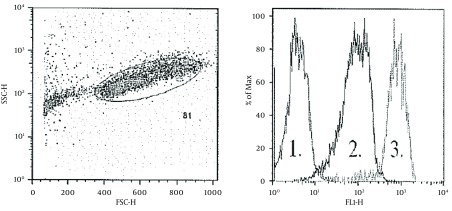
Representative Flow Cytometry Data From PBMCs. A) Gating strategy: SSC vs FSC for PBMC. B) Expression of Hsp72 in 1) unstained cells, 2) pre exercise, and 3) after 42°C incubation.

PBMC’s were separated from 1 mL of blood using density gradient centrifugation (15 min, 2100 RPM, 0 ACC) with 1.077 g/mL Histopaque (Sigma-Aldrich, St. Louis, MO). PBMC were washed with phosphate buffered saline (PBS) and then treated with Reagent A (Fix and Perm kit, Invitrogen, Carlsbad, CA) and incubated at room temperature for 15 min. Cells were then washed and treated with Reagent B (Fix and Perm kit, Invitrogen, Carlsbad, CA) combined with a monoclonal Hsp72 FITC antibody (Assay Designs, Ann Arbor, MI) at 100 μg/mL. Cells were incubated for 20 minutes in the dark at room temperature. Cells were washed a final time, and then diluted in 300 mL sheath fluid and analyzed using a FACSCAN cytometer (BD Scientific, San Jose, CA). Ten thousand events were collected. Data was analyzed using Cellquest software (BD Scientific, San Jose, CA). The amount of protein produced per cell population was quantified as mean fluorescent intensity (MFI). To determine MFI, cells were gated and corrected for auto-fluorescence based upon unstained control. The gating strategy is shown in Figure 1. 

Plasma was analyzed for estrogen and progesterone using ELISA kits (Genway Bioscience, San Diego, CA) according to manufacturer’s instruction. The minimum detectable concentration was 5 ± 2 pg/mL for estradiol and 0.08 ± 0.03 ng/mL for progesterone. Inter-assay variability was 6% for estradiol and 8.8% for progesterone. Intra-assay variability was 4.6% for estradiol and 9.7% for progesterone.

### 3.6. Statistical Analysis

Hsp72 was expressed as MFI, and, to more accurately assess sex differences due to exercise, pre exercise values were normalized to 1. Thus, the post exercise expression of Hsp72 was expressed as the change from the pre exercise value as previously reported (24, 25). A three factor (time x trial x sex) ANOVA was used to determine changes due to exercise. Sampling times included pre-exercise, immediately post exercise, 1 hr post exercise, and 4 hrs post-exercise. 

Menstrual cycle comparisons were done only for those women in whom their phase of the cycle was confirmed by hormonal analysis (see results). A two-factor (time x phase) repeated measures ANOVA was used to test for differences due to the menstrual cycle phase. Here we compared fol vs. lut data, n = 7. To determine the potential effect of ovarian hormones on Hsp72, a correlation analysis between estrogen and Hsp72 in addition to progesterone and Hsp72 was conducted for all women.

All descriptive data were analyzed using a t-test (weight, aerobic capacity, % body fat, and hormonal analysis of estrogen and progesterone). Independent t-tests were used to compare men’s and women’s values for T_r_, HR, urine osmolality, and relative exercise intensity.

The necessary *n* size was estimated to be 8 subjects, given a power of 0.80 and an alpha level of 0.05, using Statistica. Because sex differences in Hsp72 have not been assessed, we estimated *n* size according to the change from baseline in monocyte’s of males after treadmill exercise in the heat (6, 9) .

Statistical significance was set at α ≤ 0.05 and analysis was performed using Statistica 10 (StatSoft Inc, Tulsa, OK). Data were assessed for normality and homogeneity of variance prior to statistical analysis. Hsp72 data were not normally distributed and thus log transformed prior to analysis. Tukey’s post hoc test was used if necessary. Data is presented as mean ± SD in text and tables. For simplicity, data in figures is presented as mean ± SEM .

## 4. Results 

### 4.1. Subject Characteristics

Men and women differed significantly for height (P = 0.01), weight (P = 0.01), % body fat (P = 0.01), and aerobic capacity expressed as ml kg ^-1 ^ min ^-1 ^(P = 0.03). However, when aerobic capacity was expressed relative to fat free mass, there was no difference between sexes (Table 1). For men, trial 2 was repeated 12 ± 4 days after trial 1. For women, trial 2 was repeated 12 ± 2 days after trial 1.

**Table 1.** Subject Characteristics.

	Age, y	Height, cm	Weight, kg	VO2pk, ml^-1^kg^-1^min^-1^	VO2pk, ml^-1^kgFFM^-1^min-1	Body Fat, %
**Men**	26 ± 5	182 ± 7^a^	81.0 ± 15^a^	52.6 ± 8^a^	60.0 ± 7.7	12.0 ± 5.5^a^
**Women**	24 ± 3	170 ± 3	63.1 ± 12	44.9 ± 5	58.8 ± 5.2	21.4 ± 2.5

^a^Difference (P < 0.05) between sexes.

### 4.2. Exercise Response

Men and women did not differ in starting or ending T _r _, HR, pre or post urine osmolality, or relative exercise intensity. Men had significantly greater sweat rates than women (P = 0.01). One woman experienced heat illness symptoms at 45 min during trial 1, and one male subject stopped exercising for similar reasons at 30 min on trial 1. The second bout for both subjects was identical in duration and intensity to the first bout. Hsp72 levels for these subjects were not anomalously higher after the first trial compared with the second. Therefore, data from these subjects are included in the analysis. All other subjects completed 60 min of exercise. There was no significant effect of menstrual phase on baseline or exercise T _r _, urine osmolality, ending HR, or exercise intensity (Table 2). 

**Table 2.** Thermal and Cardiovascular Response^a^to Exercise (n = 9 Men and 9 Women) (Mean ± SD).

	Pre Tc (C)	End Tc (C)	Pre Urine Osm, mOsm/kg	Post Urine Osm, mOsm/kg	End HR	Mean % VO_2pk_, mL/kg/min	Sweat Rate, mL/min
**Women Trial 1**	37.21 ± 0.08	39.05 ± 0.15	499 ± 143	453 ± 105	162 ± 4	60 ± 1.3	16 ± 2
**Women Trial 2**	37.17 ± 0.06	38.95 ± 0.17	424 82	362 ± 62	160 ± 4	58 ± 1.6	17 ± 2
**Men Trial 1**	37.04 ± 0.07	39.19 ± 0.09	559 ± 72	520 ± 83	166 ± 2	57 ± 1.5	29 ± 2^b^
**Men Trial 2**	36.98 ± 0.11	39.07 ± 0.14	442 ± 91	547 ± 95	163 ± 2	58 ± 0.75	26 ± 4^b^

^a^Response to 60 min of treadmill running at 60% VO2 pk in a 42°C environment.

^b^P < 0.05 from Women's trials.

### 4.3. Menstrual Phase Hormones

Data from two subjects were removed from the menstrual cycle comparison after examining their progesterone values. In these subjects, progesterone values were not higher in the lut compared to fol phase. Therefore, all menstrual phase analyses are shown with *n = 7 *. Of the seven subjects analyzed for menstrual phase differences, five completed their first exercise trial in the fol phase, and two subjects exercised in the lut phase first. Estrogen and progesterone levels were significantly higher (P = .01) in the lut compared to fol phase (Table 3). 

**Table 3.** Sex Hormones and Menstrual Cycle Phase (n = 7) (mean ± SD).

	Estrogen, pmol/L	Progesterone, nmol/L	Ending HR	% VO_2pk_	Pre Tc (C)	End Tc (C)
**Follicular**	344 ± 95^a^	3.35 ± 1.2^a^	160 ± 7.1	56 ± 0.02	37.19 ± 0.16	39.01 ± 0.53
**Luteal**	436 ± 89	30.1 ± 13.9	163 ± 7.0	58 ± 0.01	37.35 ± 0.28	39.06 ± 0.44

^a^P < 0.05 from Lut

### 4.4. Hsp72 Exercise Response

**Figure 2.**
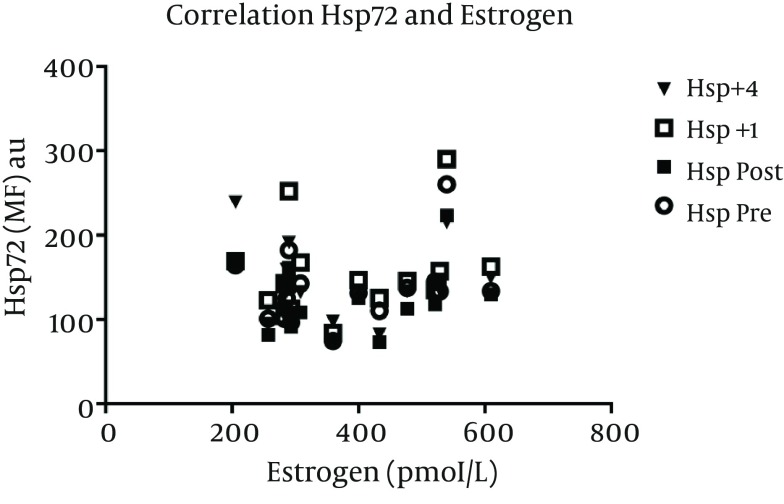
Normalized Hsp72, Exercise Responses Hsp72 response to 60 min of treadmill running at 60% VO2pk in a 42°C environment, as a percentage of baseline values. Dark bars represent men, open bars represent women. The dashed line separates trial 1 from trial 2. There was a time x trial x sex interaction: * P < 0.05 from pre exercise values for men trial 1. and P < 0.05 from trial 1 at the same time points for men. + P < 0.05 from women pre and post exercise during trial 1. Data represent mean ± SEM.

There was no difference in pre exercise values of Hsp72 MFI between sexes on trial 1 (men: 111±30, women: 121 ± 34) or 2 (men: 145 ± 38, women: 151 ± 43). The normalized, overall Hsp72 response after exercise was greater in men than women (time x trial x sex interaction P = 0.01) (Figure 2). Men expressed higher Hsp72 at +1 and +4 hrs post exercise compared to women pre and post during trial 1. However, this sex difference did not occur during trial 2. While the normalized increase in Hsp72 from pre exercise values was not significant in women during either trial, men increased Hsp72 at +1 and +4 hrs from pre exercise values in trial 1 (time x sex interaction P = 0.05). Both men and women expressed less Hsp72 in trial 2 compared to trial 1 (time x trial interaction P =<0.001). 

### 4.5. Hsp72 Exercise Response – Menstrual Phase

**Figure 3.**
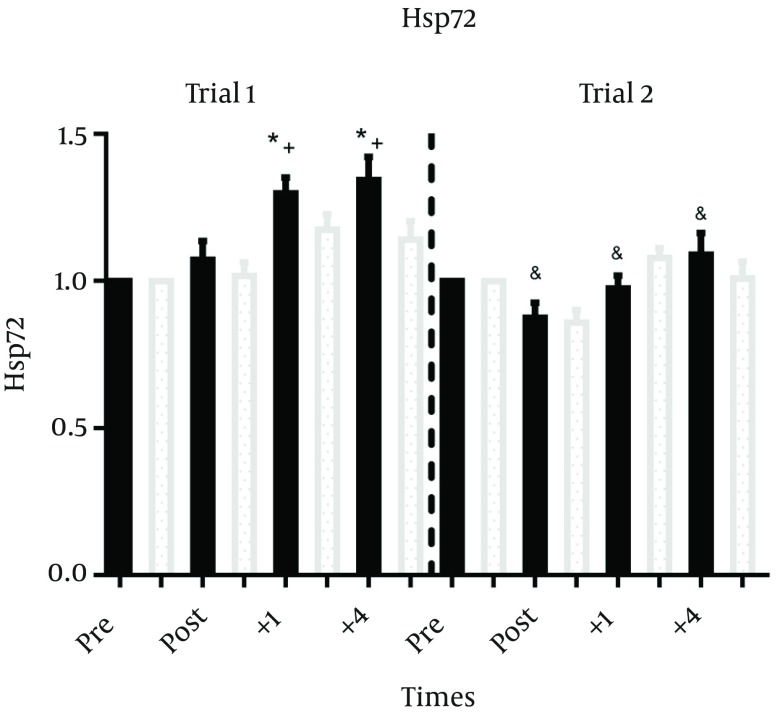
Menstrual Phase Hsp72 response to 60 min of treadmill running at 60% VO2pk in a 42°C environment, as a percentage of baseline values. Closed bars represent follicular phase, open bars represent luteal phase (n = 7). There was a main effect of time: * P < 0.05 from pre values. Data represents mean ± SEM.

**Figure 4.**
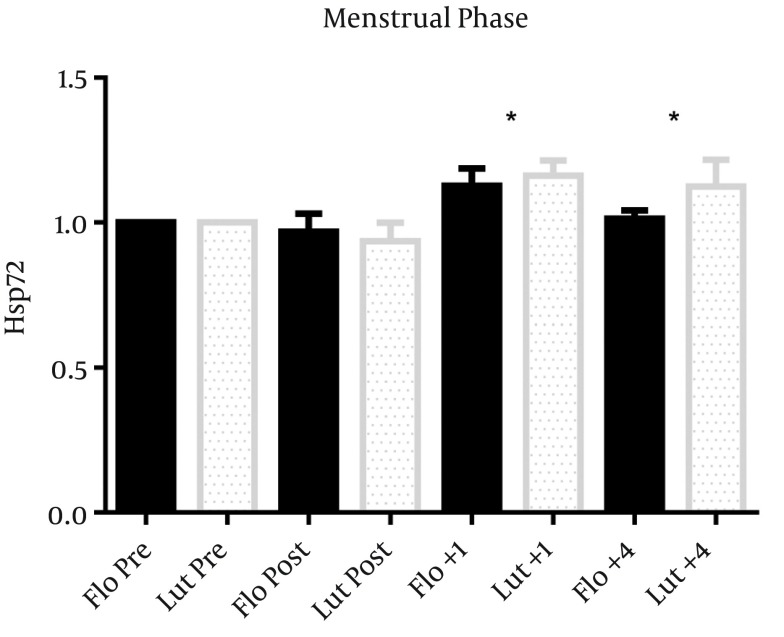
Correlation between Estrogen and Hsp72. Pre exercise estrogen concentration was not correlated with Hsp72 at any time point. Data represents n = 9.

**Figure 5.**
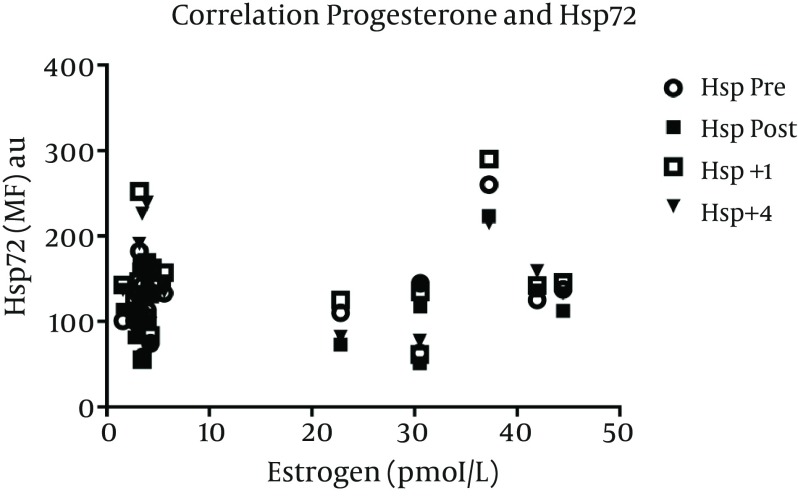
Correlation Between Progesterone and Hsp72. Pre exercise progesterone concentration was not correlated with Hsp72 at any time point. Data represents n = 9.

In the 7 women included in the menstrual phase analysis, there was no difference in pre exercise values of Hsp72 MFI expression in fol (117 ± 14) versus lut (123 ± 24) phases. The normalized, overall Hsp72 response increased after exercise (main effect of time, P = 0.01), but was not different between fol and lut phases (Figure 3). For all female subjects, estrogen (r = 0.24, 0.12, 0.17, -0.7) and progesterone (r = 0.27, 0.13, 0.14, -0.1) concentrations were not correlated to Hsp72 MFI values at pre, post, +1, and +4 hrs post exercise, respectively (Figures 4 and 5).

## 5. Discussion

Our major finding is that men and women have different Hsp72 stress responses to exercise in the heat. Men expressed greater Hsp72 after exercise in trial 1 than women, and subsequently up-regulated their baseline Hsp72 expression by ~30% before trial 2. This increase in baseline Hsp72 led to reduced Hsp72 expression when the same exercise stress was repeated 12 days later. Women produced less Hsp72 than men on trial 1 and did not up regulate baseline Hsp72 expression during trial 2. Thus, in response to an initial exercise challenge, women had a blunted cellular stress response compared to men. This sex difference may highlight the redundant mechanisms of estrogen and Hsp72 in mediating the stress response to exercise in the heat. Estrogen may help to stabilize cell membranes, thus reducing the need to up regulate Hsp72 after an acute stress. Thus, we reject our hypothesis that women would have an increased pre exercise expression of Hsp72, but retain our hypothesis that the post exercise response in women would be attenuated compared to men. In addition, there was no effect of menstrual phase either at rest or after exercise on Hsp72 expression. Therefore, we reject our hypothesis that during lut, when estrogen levels are elevated, there would be a decrease in Hsp72 at rest and after exercise compared to fol.

Hsp72 expression has a significant role in maintaining cellular homeostasis during and after stress (4, 26). While numerous animal studies have found sex differences in Hsp72 expression with acute hyperthermia or exercise, to our knowledge this is the first study to examine this effect in humans. Results from animal studies imply that estrogen is responsible for increased baseline Hsp72 expression (14, 15, 23) and this up-regulation exerts protective effects that reduce the need for Hsp72 production during exercise (16, 19), ischemia (17), or hyperthermic (18) stresses. In our study in humans, the baseline Hsp72 did not differ between sexes. However, men expressed greater Hsp72 compared to women in response to stress. Thus, while it is understood that male animals increase Hsp72 to a greater extent in response to stress than females, we report for the first time that this sex effect is also evident in humans. 

Acquired cellular thermotolerance occurs when a single exposure to a severe, but sub-lethal heat stress leads to protection against future, more severe heat exposure. This process involves the increased expression of basal Hsp72 (2, 3) and leads to decreased Hsp72 induction in response to a second exposure (24). In this fashion, Hsp72 can act as a marker for thermal history (10). In men, we found a greater percent increase in baseline PBMC Hsp72 content 12 days after trial 1 compared to women. Although this was not statistically significant, it is likely that the increased baseline expression seen in men in trial 2 abrogated the need for further Hsp72 production during trial 2. As such, neither men nor women showed a significant accumulation of Hsp72 in PBMCs during the second trial. Thus, it appears that Hsp72 may be regulated differently after an acute bout of exercise in the heat in non- heat acclimated men and women. It has not been previously appreciated that baseline Hsp72 could be up-regulated 12 days after a single acute bout of exercise in the heat. This data raises interesting questions regarding the role of thermotolerance in women. Estrogen may relegate the process of acquired cellular thermotolerance redundant, diminishing the need for Hsp72 accumulation. 

This paper also is the first to examine possible effects of the menstrual cycle on Hsp72 expression. If estrogen is responsible for the decreased Hsp72 induction during exercise, then the variation of estrogen across the menstrual cycle could alter baseline or stress induced Hsp72 expression. The mechanism behind the relationship between estrogen and the blunted intracellular Hsp72 response to stress currently is not known. It was suggested that estrogen mediates this effect through a nongenomic hormonal pathway. Treating animals with tamoxifen, a known estrogen receptor agonist, caused the same blunted post exercise Hsp70 expression as in ovariectomized animals treated with 17β and 17α estradiol (17). Since tamoxifen, 17β, and 17α estradiol all suppress the post exercise expression of Hsp70, researchers suggest that these estrogen related compounds stabilize cell membranes and attenuate oxidative stress (25). Such an effect could protect thermal sensitive cells against exercise-induced damage, and thereby result in a blunted Hsp72 expression. However, we found no difference in baseline or exercise Hsp72 response when women exercised in the fol compared to the lut phase. While our statistical power was limited with only 7 subjects included, there was no correlation between ovarian hormone concentrations and Hsp72 expression with *n = 9*. Our findings are supported by animal data that suggest no effect of the estrous cycle on Hsp72 production in the pituitary or adrenal gland, spleen, lymph nodes, liver or heart in response to stress (27). Thus, the physiologic variations in estrogen and progesterone during the menstrual cycle may not be sufficient to alter Hsp72 expression. 

Our main finding is that non-acclimated men increased Hsp72 more than women in PBMCs in response to exercise in the heat. After this single bout of exercise, men’s Hsp72 was up-regulated for up to 12 days, suggesting that men had acquired cellular thermal tolerance. We suggest that estrogen may provide cellular protection and thus decrease the need to up-regulate Hsp72 in non-acclimated women. These data raise intriguing questions about the role of acquired cellular thermal tolerance between sexes.
